# Adjuvant chemotherapy in pathological node-negative non-small cell lung cancer

**DOI:** 10.1038/s41598-023-46679-8

**Published:** 2023-11-06

**Authors:** Ya-Fu Cheng, Yi-Ling Chen, Chia-Chi Liu, Ching-Min Lin, Shao-Syuan Tong, Bing-Yen Wang

**Affiliations:** 1 Division of Thoracic Surgery, Department of Surgery, Changhua Christian Hospital, No. 135 Nanxiao St., Changhua City, Changhua County 500 Taiwan; 2 Surgery Clinical Research Center, Changhua Christian Hospital, Changhua, Taiwan; 3 Department of Post-Baccalaureate Medicine, College of Medicine, National Chung Hsing University, Taichung, Taiwan

**Keywords:** Cancer, Medical research, Oncology

## Abstract

Non–small cell lung cancer (NSCLC) is associated with a poor survival rate, even for patients with early-stage cancer. Identifying patients with pathological N0 NSCLC who may benefit from adjuvant chemotherapy treatment after surgery is essential. We conducted a retrospective cohort study used data from the Surveillance, Epidemiology, and End Results database and included 26,380 patients with pathological N0 NSCLC after surgery between January 2018, and December 2019. Among 26,380 patients, 24,273 patients received surgery alone and the other 2107 patients received surgery plus adjuvant chemotherapy. After 1:1 propensity score matching, both groups contained 2107 patients. Adjuvant chemotherapy did not show significantly better 24-month survival in T2aN0 NSCLC patients (83.41% vs. 82.91%, p = 0.067), although it did for T2bN0 patients (86.36% vs. 81.70%, p = 0.028). Poorly-differentiated NSCLC remained a high-risk factor for pT2N0, and adjuvant chemotherapy provided better 24-month survival after matching (86.36% vs. 81.70%, p = 0.029). In conclusion, when treating pN0 NSCLC, adjuvant chemotherapy had a beneficial effect when the tumor size was larger than 4 cm. The effect when the tumor size was between 3 and 4 cm was not remarkable. Poorly-differentiated NSCLC was a high-risk factor in the pT2N0 stage.

## Introduction

Lung cancer is identified as the leading cause of cancer death in both genders worldwide^1^. Around 85% of lung cancers are identified as non–small cell lung cancer (NSCLC). The survival rate still remains poor, even for patients with early-stage NSCLC. There is only a 75% 5-year overall survival (OS) rate for patients with a pathological N0 nodal stage^2^. Although complete surgical resection is vital to improve the survival rate in these patients, identifying patients with pathological N0 NSCLC who may benefit from adjuvant chemotherapy treatment after surgery is also essential^3^.

The National Comprehensive Cancer Network (NCCN) guidelines suggest that adjuvant chemotherapy should be applied in all patients with pathological N1 and N2 NSCLC^4^. Using adjuvant chemotherapy should be carefully considered due to its toxicity and immunosuppressive effects. In patients with pathological N0 NSCLC, adjuvant chemotherapy is not recommended when the tumor status is T1a to T1c, but it may have benefits at the T2a (3.1 cm to 4.0 cm) and T2b (4.1 cm to 5.0 cm) statuses when there is a high-risk factor such as a poorly-differentiated tumor, vascular invasion, wedge resection, visceral pleural involvement or unknown lymph node status. However, these recommendations are based on low levels of evidence and little supporting data^5^. The treatment effects of adjuvant chemotherapy in these high-risk patients are still unclear.

Traditionally, most large randomized trials considered tumor size alone. More recently, the pairings of tumor size with several high-risk factors have been proposed as more accurate indications for adjuvant chemotherapy in early-stage NSCLC patients^3^. Previous studies on the effectiveness of adjuvant chemotherapy used data based on 6th or 7th edition of the lung cancer staging system from the American Joint Committee on Cancer (AJCC) and converted the data to fit the 8th edition. That data might be too old to reflect current treatment statuses since surgical procedures have rapidly evolved and improved in recent years. Therefore, this study aimed to find out the effects of adjuvant chemotherapy for pathological N0 NSCLC patients with current data that reflects the 8th edition of the AJCC’s lung cancer staging system.

## Patients and Methods

### Patient population and selection

Population data were obtained from the Surveillance, Epidemiology, and End Results (SEER) database supported by the Surveillance Research Program in the Division of Cancer Control and Population Sciences of the National Cancer Institute in the US. All the details and data were obtained from the SEER public access database using version 8.4.0.1 of the SEER*Stat software. We collected information on NSCLC patients who were diagnosed between 2018 and 2019. Thus, the information was based on the 8th edition of the AJCC’s lung cancer staging system. We included pathological N0 patients who received surgical intervention. Patients were excluded if they were younger than eighteen years old, had a T0/Tis/Tmi status, had an unspecified histology or received systemic therapy before surgery. Patients were also excluded if their database records were missing values for therapy or T status. Our study was approved by our institutional review board (IRB-210808) (Changhua Christian Hospital, Changhua, Taiwan). Informed consent from all participants was waived with the understanding that the released information would be used strictly for research purposes.

The following patient characteristics were collected from the SEER database: age, gender, tumor characteristics (histologic grade, cell type, and T stage), treatment details (chemotherapy and radiotherapy) and surgical method. The histologic subtypes were classified into adenocarcinoma (AD), squamous cell carcinoma (SqCC), and other histologic types. The outcome measure for our study was 24-month OS rate. OS was calculated as the time from date of diagnosis to either death from any cause or the 2020 cutoff.

### Statistical analyses

We examined two groups of patients: those that had surgery alone and those that had surgery plus adjuvant chemotherapy. Propensity score matching was used with age, gender, cell type and T status to minimize the bias. Histology grade could not be used in the matching due to there being too few patients with undifferentiated tumors. We used Wilcoxon rank-sum tests for continuous variables and Chi-squared or Fisher’s exact tests for categorical variables. Survival curves were plotted using the Kaplan–Meier method. Univariate and multivariate analyses were performed using a Cox proportional hazards regression model. The following clinical-pathologic factors were included in the analyses: age, gender, histologic grade, cell type, T stage, therapeutic method and surgical method. All calculations were performed using IBM SPSS Statistics for Windows, version 22.0 (IBM Corp., Armonk, NY). Statistical analysis with a p-value less than 0.05 was considered statistically significant.

### Ethics approval

This is an observational study. The institutional review board in our institute has confirmed that no ethical approval is required.

## Results

Data from 31,545 patients with NSCLC were analyzed. Based on the above exclusion criteria, 5165 patients were excluded. The remaining 26,380 patients were included in this study. There were 24,273 patients who received surgery alone, and the other 2107 patients received surgery plus adjuvant chemotherapy. The detailed baseline characteristics were shown in Table 1. All the variables were significantly different between the two groups.

**Table 1:** Baseline characteristics before matching.

	Surgery alone (n = 24,273)	Surgery plus adjuvant chemotherapy (n = 2107)	p-value
Age in years (mean ± SD)	71.25 ± 9.80	67.17 ± 8.44	< 0.001
Sex			
Male	10,885 (44.8%)	1055 (50.1%)	< 0.001
Female	13,388 (55.2%)	1052 (49.9%)	
Histologic grade			
Well differentiated	2592 (10.7%)	147 (7.0%)	< 0.001
Moderately differentiated	5252 (21.6%)	632 (30.0%)	
Poorly differentiated	2497 (10.3%)	595 (28.2%)	
Undifferentiated	51 (0.2%)	43 (2.0%)	
Unknown	13,881 (57.2%)	690 (32.7%)	
Cell type			
Adenocarcinoma	12,592 (51.9%)	889 (42.2%)	< 0.001
Squamous cell carcinoma	6540 (26.9%)	626 (29.7%)	
Others	5141 (21.2%)	592 (28.1%)	
T status			
T1a	2336 (9.6%)	68 (3.2%)	< 0.001
T1b	7561 (31.1%)	164 (7.8%)	
T1c	4701 (19.4%)	121 (5.7%)	
T2a	4586 (18.9%)	454 (21.5%)	
T2b	1200 (4.9%)	275 (13.1%)	
T3	2065 (8.5%)	609 (28.9%)	
T4	1824 (7.5%)	416 (19.7%)	
Surgical methods			
Wedge resection	2376 (9.8%)	208 (9.9%)	< 0.001
Segmentectomy	900 (3.7%)	63 (3.0%)	
Lobectomy/bilobectomy	8818 (36.3%)	1323 (62.8%)	
Others or unknown	12,179 (50.2%)	513 (24.3%)	

We used 1:1 propensity score matching to reduce the treatment assignment bias. Both groups contained 2107 patients after matching (Table 2). The mean age was approximately 67 years in each group. In both groups, there were roughly as many males as females. Due to the miniscule number of undifferentiated tumors, we could not match by histologic grade in this study. Patients who received surgery plus adjuvant chemotherapy had higher ratios of moderately-differentiated (*n* = 632, 30.0% vs. *n* = 619, 29.4%), poorly-differentiated (*n* = 595, 28.2% vs. *n* = 375, 17.8%) and undifferentiated (*n* = 43, 2.0% vs. *n* = 11, 0.5%) tumors. There were 919 patients (43.6%) with AD, 631 patients (29.9%) with SqCC and 557 patients (26.4%) with other cell types in the surgery alone group, while there were 889 patients (42.2%) with AD, 626 patients (29.7%) with SqCC and 592 patients (28.1%) with other cell types in the surgery plus adjuvant chemotherapy group. The T statuses were equally distributed in both groups with T2 statuses approximately accounting for 34% of the cases and merely 17% of patients having a T1 status. Most patients received lobectomy/bi-lobectomy in both groups (around 60%). Wedge resection and segmentectomy were in a tiny minority group.

**Table 2:** Baseline characteristics after matching.

	Surgery alone (n = 2107)	Surgery plus adjuvant chemotherapy (n = 2107)	p-value
Age in years (mean ± SD)	67.64 ± 8.19	67.17 ± 8.44	0.065
Sex			
Male	1015 (48.2%)	1055 (50.1%)	0.218
Female	1092 (51.8%)	1052 (49.9%)	
Histologic grade			
Well differentiated	306 (14.5%)	147 (7.0%)	< 0.001
Moderately differentiated	619 (29.4%)	632 (30.0%)	
Poorly differentiated	375 (17.8%)	595 (28.2%)	
Undifferentiated	11 (0.5%)	43 (2.0%)	
Unknown	796 (37.8%)	690 (32.7%)	
Cell type			
Adenocarcinoma	919 (43.6%)	889 (42.2%)	0.453
Squamous cell carcinoma	631 (29.9%)	626 (29.7%)	
Others	557 (26.4%)	592 (28.1%)	
T status			
T1a	68 (3.2%)	68 (3.2%)	0.999
T1b	173 (8.2%)	164 (7.8%)	
T1c	122 (5.8%)	121 (5.7%)	
T2a	456 (21.6%)	454 (21.5%)	
T2b	271 (12.9%)	275 (13.1%)	
T3	606 (28.8%)	609 (28.9%)	
T4	411 (19.5%)	416 (19.7%)	
Surgical methods			
Wedge resection	214 (10.2%)	208 (9.9%)	0.235
Segmentectomy	61 (2.9%)	63 (3.0%)	
Lobectomy/bilobectomy	1264 (60.0%)	1323 (62.8%)	
Others or unknown	568 (27.0%)	513 (24.3%)	

**Figure 1:**
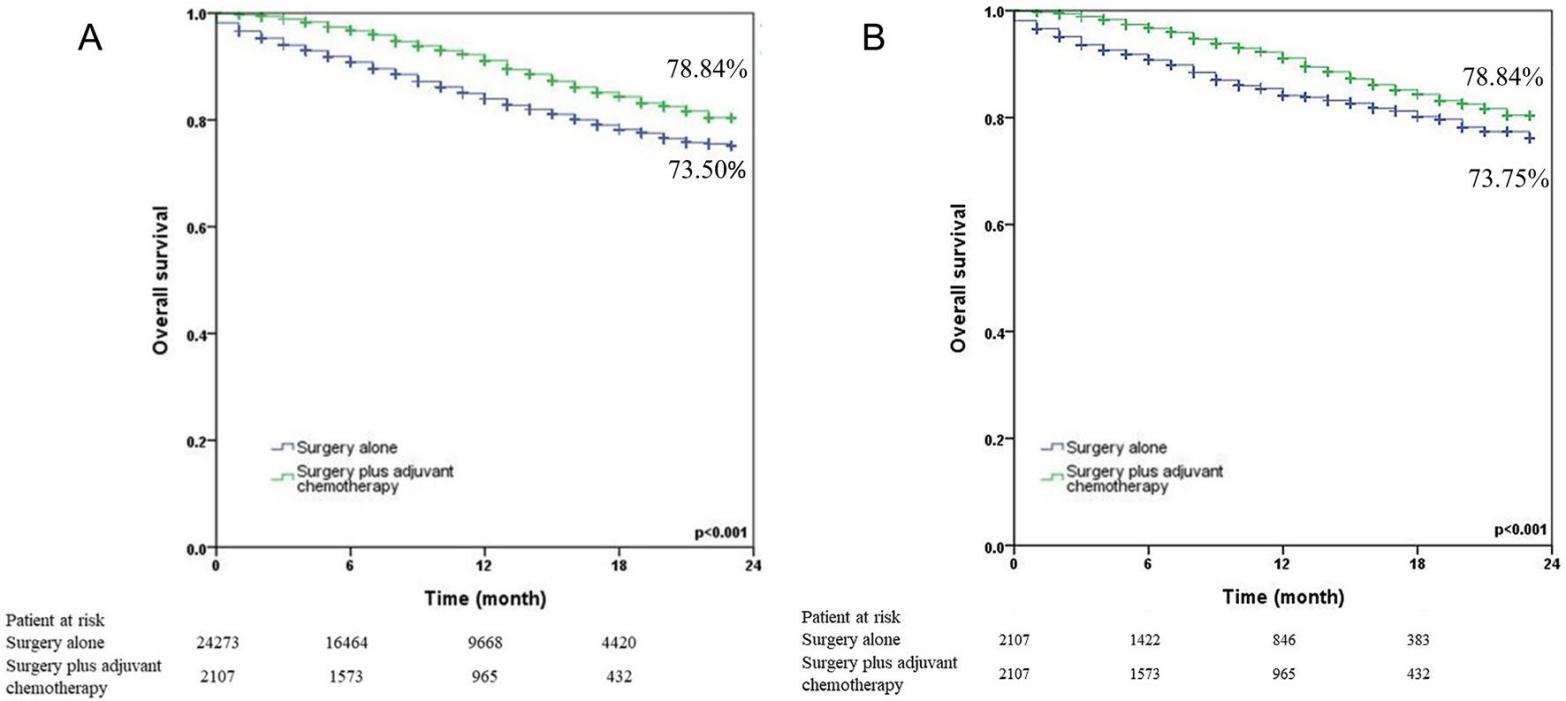
Kaplan–Meier 24-month survival curves by treatment method for N0 NSCLC patients (**A**) before propensity matching (**B**) after propensity matching.

**Figure 2:**
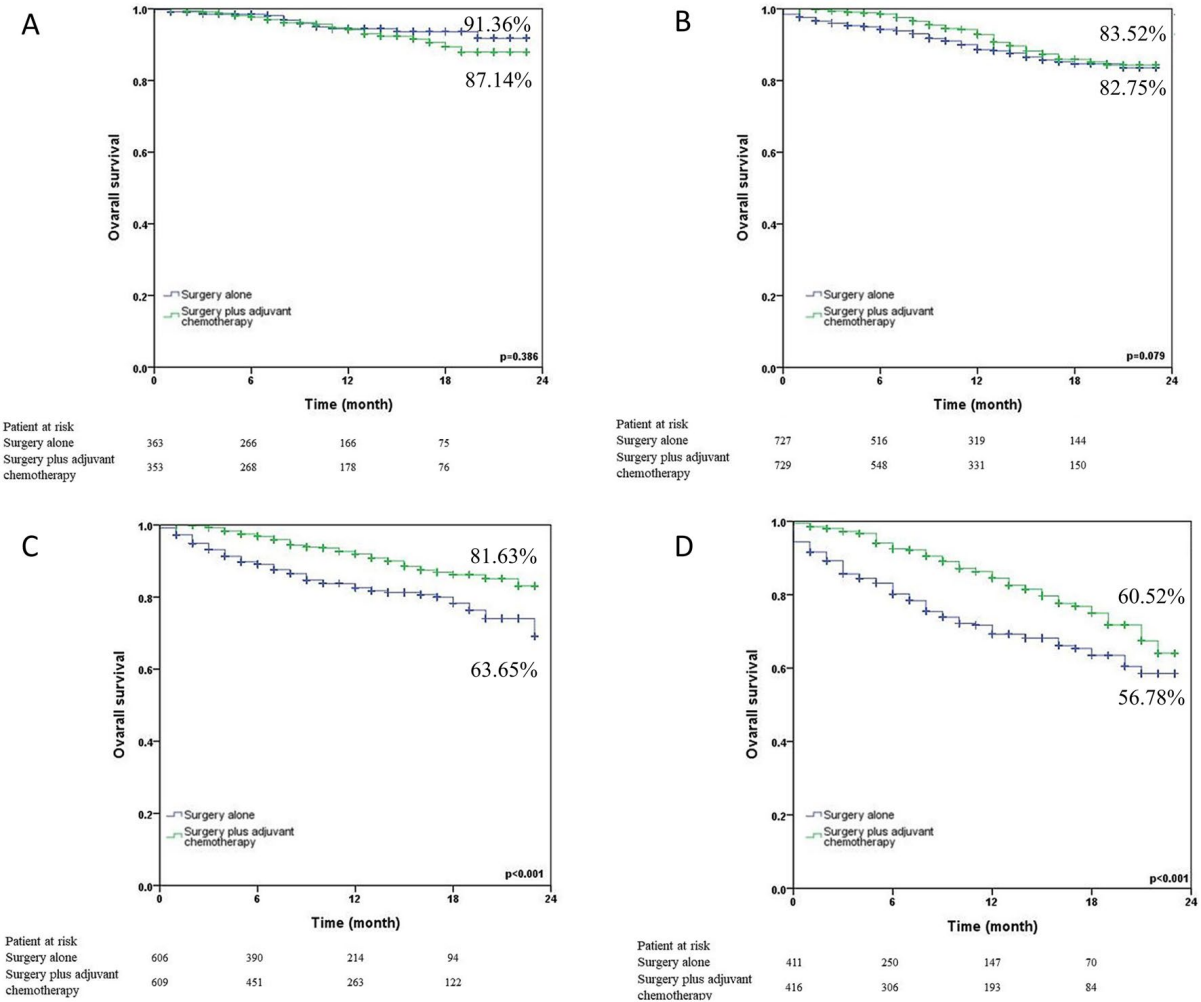
Kaplan–Meier 24-month survival curves by T status and treatment method for N0 NSCLC patients after propensity matching (**A**) T1N0 (**B**) T2N0 (**C**) T3N0 (**D**) T4N0.

**Figure 3:**
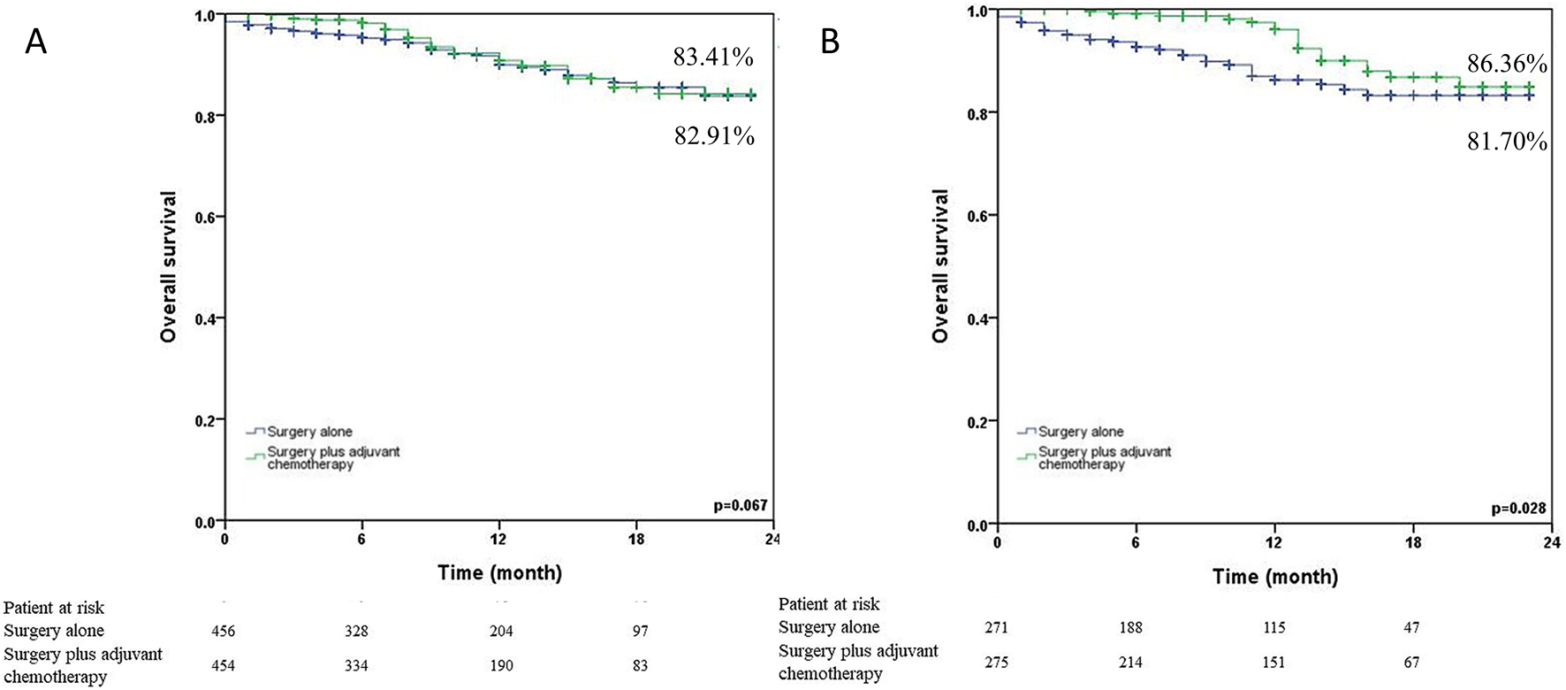
Kaplan–Meier 24-month survival curves by treatment method for T2N0 NSCLC patients after propensity matching (**A**) T2aN0 (**B**) T2bN0.

The 24-month survival curves for the surgery alone group and the surgery plus adjuvant chemotherapy group were shown in Fig. 1 (1a: before matching; 1b: after matching). Surgery plus adjuvant chemotherapy showed significantly better 24-month survival than surgery alone in N0 NSCLC patients both before (78.84% vs. 73.50%, p < 0.001) and after matching (78.84% vs. 73.75%, p < 0.001). We also analyzed the 24-month survival curves by T status (Fig. 2). Surgery plus adjuvant chemotherapy did not reveal significantly better 24-month survival than surgery alone in the T1 status (87.14% vs. 91.36%, p = 0.386) and T2 (83.52% vs. 82.75%, p = 0.079). On the other hand, surgery plus adjuvant chemotherapy provided a benefit in the T3 (81.63% vs. 63.65%, p < 0.001) and T4 (60.52% vs. 56.78%, p < 0.001) statuses. Figure 3 illustrated the subgroup analysis of T2a and T2b NSCLC patients after propensity matching. Surgery plus adjuvant chemotherapy did not show significantly better 24-month survival than surgery alone in T2aN0 NSCLC patients (83.41% vs. 82.91%, p = 0.067), but it did show improved survival in T2bN0 patients (86.36% vs. 81.70%, p = 0.028).

**Figure 4:**
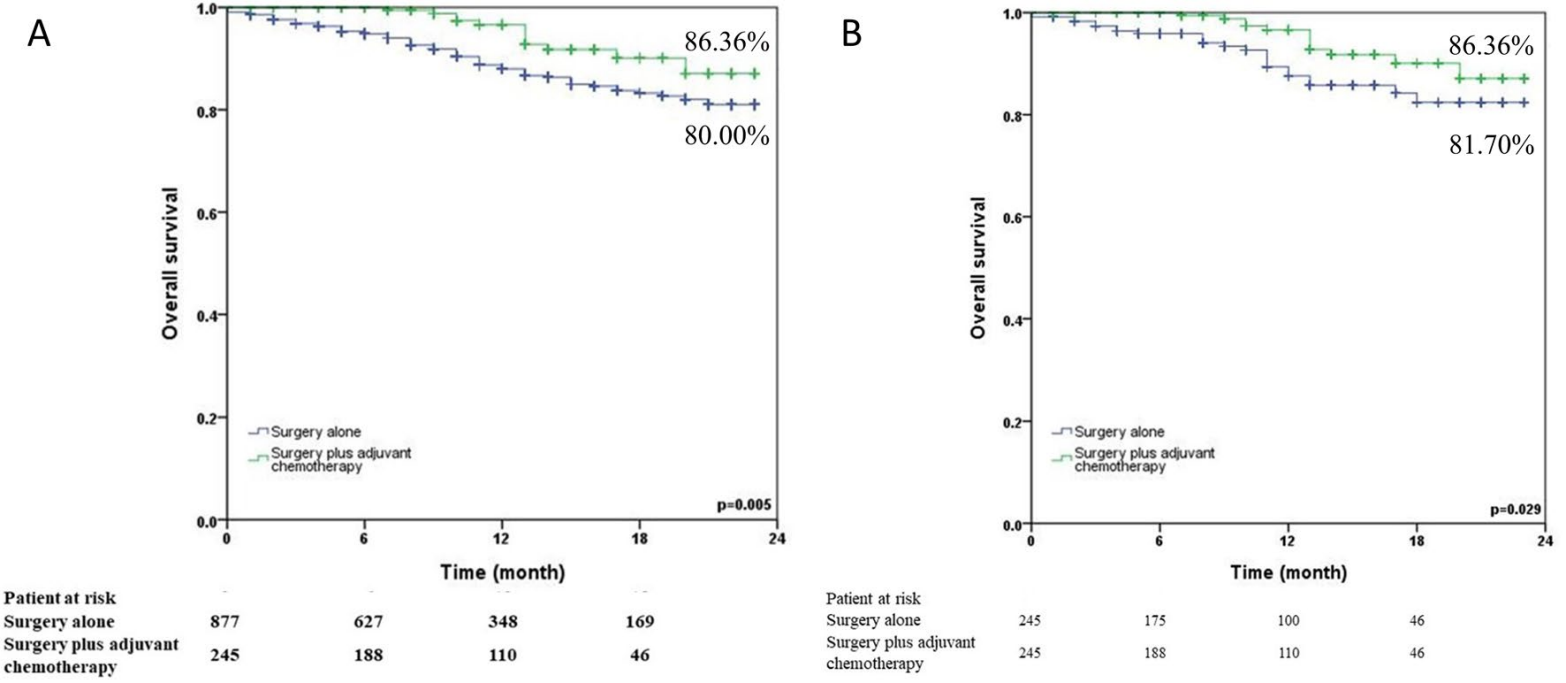
Kaplan–Meier 24-month survival curves by treatment method for poorly-differentiated T2N0 NSCLC patients (**A**) before propensity matching (**B**) after propensity matching.

Whether a poorly-differentiated tumor is a high-risk factor in T2N0 NSCLC patients still remains controversial. We did a subgroup analysis of these patients in which 877 patients received surgery alone and 245 patients received surgery plus adjuvant chemotherapy (Fig. 4). Surgery plus adjuvant chemotherapy provided significantly better 24-month survival than surgery alone both before (86.36% vs. 80.00%, p = 0.005) and after matching (86.36% vs. 81.70%, p = 0.029).

Both univariate and multivariate Cox regression models were analyzed (Table 3). In univariate analysis, young age, female gender, well-differentiated histologic grade, AD cell type, early T stage and received adjuvant chemotherapy were statistically associated with better 24-month survival rates. Similar trends were also found in multivariate analysis.

**Table 3:** Univariate and multivariate analyses of overall survival before matching.

Variables	Univariate analysis			Multivariate analysis		
	HR	95% CI	p-value	HR	95% CI	p-value
Age in years	1.04	1.03 ~ 1.05	< 0.001	1.03	1.02 ~ 1.03	< 0.001
Gender						
Male (reference)	1	–		1		
Female	0.54	0.46 ~ 0.65	< 0.001	0.75	0.71 ~ 0.81	< 0.001
Grade						
Well (reference)	1			1		
Moderately	2.91	1.49 ~ 5.68	0.002	1.88	1.45 ~ 2.43	< 0.001
Poorly differentiated	3.20	1.63 ~ 6.28	0.001	2.54	1.95 ~ 3.31	< 0.001
Undifferentiated	4.74	1.64 ~ 13.70	0.004	3.38	1.79 ~ 6.41	< 0.001
Unknown	10.67	5.64 ~ 20.18	< 0.001	1.87	1.44 ~ 2.43	< 0.001
Cell type						
AD	1			1		
SqCC	2.24	1.84 ~ 2.73	< 0.001	1.46	1.36 ~ 1.28	< 0.001
Others	1.20	0.96 ~ 1.50	0.112	1.55	1.41 ~ 1.69	< 0.001
T status						
T1a (reference)	1			1		
T1b	0.59	0.28 ~ 1.26	0.174	1.14	0.95 ~ 1.37	0.150
T1c	0.76	0.35 ~ 1.64	0.481	1.38	1.15 ~ 1.66	0.001
T2a	1.06	0.56 ~ 2.00	0.851	2.06	1.72 ~ 2.46	< 0.001
T2b	1.60	0.84 ~ 3.05	0.150	2.95	2.43 ~ 3.60	< 0.001
T3	1.87	1.01 ~ 3.46	0.045	3.06	2.55 ~ 3.67	< 0.001
T4	3.96	2.15 ~ 7.29	< 0.001	4.50	3.76 ~ 5.39	< 0.001
Surgical methods						
Wedge resection	1					
Segmentectomy	0.73	0.51 ~ 1.05	0.099	0.79	0.55 ~ 1.14	0.204
Lobectomy/bilobectomy	0.89	0.74 ~ 1.07	0.203	0.82	0.68 ~ 0.98	0.033
Others or unknown	4.90	4.15 ~ 5.80	< 0.001	3.40	2.79 ~ 4.16	< 0.001
Adjuvant chemotherapy						
Yes (reference)	1			1		
No	2.60	2.17 ~ 3.11	< 0.001	1.86	1.60 ~ 2.16	< 0.001

*HR* hazard ratio, *CI* confidence interval, *AD* adenocarcinoma, *SqCC* squamous cell carcinoma.

## Discussion

This study is a retrospective investigation of the treatment effect of adjuvant chemotherapy in pathological N0 NSCLC patients. We suggest that adjuvant chemotherapy has a benefit for such patients when their T status is T2b or higher. The effect for stage T2aN0 is not remarkable. Poorly-differentiated NSCLC is still a high-risk factor in stage T2N0, and adjuvant chemotherapy is recommended in such cases.

The effect of adjuvant chemotherapy on pathological N0 NSCLC tumors between 3 and 5 cm in size has been controversial. The debate has changed as chemotherapy drugs and surgical methods have evolved. The original randomized control trial dates back to CALGB 9633^6^. A statistically significant survival advantage was noted for 344 patients with tumors larger than 4 cm who had adjuvant paclitaxel/carboplatin. On the contrary, a later JBR-10 trial reported that adjuvant chemotherapy did not provide a survival benefit for pathological N0 NSCLC patients with a tumor size between 3 and 5 cm^7^. The regimen of vinorelbine/cisplatin could be used in pathological N1 NSCLC. Other large randomized control trials like the ALPI, IALT, BLT and ANITA trials combined stage I to IIIA patients and did not focus on the effect of adjuvant chemotherapy^8–12^. Based on these trials during the late 2000s, tumor size has become a common single criterion for the use of adjuvant chemotherapy on pathological N0 NSCLC patients^13^. The recent JCOG 0707 trial demonstrated the benefit of tegafur-uracil (UFT) in N0 NSCLC^14^. It suggested that for tumors without ground-glass attenuation and size greater than 3 cm, patients with adjuvant UFT therapy had significantly longer survival than those without.

In developing countries, most pathological N0 NSCLC patients with tumors measuring 3 to 5 cm cannot afford adjuvant target therapy or immunotherapy. They are left with the choice of adjuvant chemotherapy or no further treatment after operation. Several recent retrospective studies have reviewed adjuvant chemotherapy outcomes in these patients. Some studies supported that adjuvant chemotherapy should be applied when the tumor size is 3 to 5 cm^15–17^. Other studies focused on a tumor size of 3 to 4 cm and suggested a positive effect^18–20^. On the contrary, others claimed that adjuvant chemotherapy did not have a positive effect on disease-free survival or OS in stage IB NSCLC patients, even for patients with multiple risk factors^21,22^. All of these studies converted data based on the 7th edition of the AJCC’s lung cancer staging system to fit analyses based on the 8th edition, which might introduce some bias and indicates that the data is old. Our study is the first study to analyze the effects of adjuvant chemotherapy on pathological N0 NSCLC patients based solely on the 8th edition of the AJCC’s lung cancer staging system. This data can best reflect the current treatments of these patients. We suggest that adjuvant chemotherapy should be applied when the tumor size is 4 to 5 cm, and it could be applied when the tumor is 3 to 4 cm in size and has a poorly-differentiated histologic grade.

In recent research, tumor size alone was not associated with improved efficacy of adjuvant chemotherapy in patients with early-stage NSCLC^3^. Improved efficacy occurs when tumor size is considered along with high-risk factors such as poorly-differentiated tumors, vascular invasion, wedge resection, visceral pleural involvement and unknown lymph node status^4^. A tumor size > 4 cm did not appear on the list of high-risk factors found in NCCN guidelines released in 2023. However, several studies provided evidence against the idea of these high-risk factors. Moon et al. reported that lymphovascular invasion was the only prognostic factor identified in patients with stage IB NSCLC^23^. Choi et al. suggested that visceral pleural invasion and vascular invasion are more relevant to OS and adjuvant therapy should be given even when the tumor size is less than 3 cm^24^. A previous meta-analysis reported that visceral pleural invasion and tumor size should be taken into account simultaneously. Patients with tumors larger than 3 cm with visceral pleural invasion might be considered for adjuvant chemotherapy^25^. We believe lymphovascular invasion and visceral pleural involvement still remain high-risk factors and highly recommended adjuvant chemotherapy for these patients.

As for patients with poorly-differentiated tumors, the concept was proposed two decades ago that they had a high recurrence rate and unfavorable prognosis^26^. A retrospective study including 179 patients with resected pathological stage I NSCLC supported that adjuvant chemotherapy might be beneficial in these patients^27^. However, more recent studies did not find the treatment benefit of adjuvant chemotherapy in poorly-differentiated tumors^23,24,28^. We did a subgroup analysis of pathological N0 NSCLC patients who had poorly-differentiated tumors measuring 3 to 5 cm. We suggest that adjuvant chemotherapy still plays a positive effect in these patients.

Regarding surgical methods, there were seldom trials that compared lobectomy to sublobar resection in pathological N0 NSCLC patients with a tumor size between 3 and 5 cm. Lobectomy still remained the standard treatment in early-stage NSCLC. The recent large randomized control trials JCOG 0802 and CALGB 140583 both supported that wedge resection and segmentectomy do not provide inferior survival compared to lobectomy for pathological N0 NSCLC when the tumor size is less than 2 cm^29,30^. No study compared sublobar resection to lobectomy when the tumor size was larger than 2 cm. Thus, patients who received sublobar resection in these groups may have contributed to selection bias pertaining to poorer comorbidities, performance status, and pulmonary function and might have less OS initially. A prospective randomized control trial is needed to determine the risk level for sublobar resection when the tumor size is larger than 2 cm.

A strength of our study is that it includes only data from after the 2017 release of the 8th edition of the AJCC’s lung cancer staging system. In addition, we analyzed T2a (tumor size: 3 to 4 cm) and T2b (tumor size: 4 to 5 cm) NSCLC separately since there have been recent disputes concerning tumor sizes > 4 cm. However, there were several limitations of our study. First, the retrospective design may contribute to selection bias, which could affect the results. Second, the study period was limited to data available between 2018 and 2019, so we were limited to 24-month OS. A future study with an observation period allowing for to 5-year or 10-year OS should be performed. Third, the SEER database did not provide detailed data on tumor subtypes and invasion status. We did not evaluate other high-risk factors about these patients, which might have caused a selection bias. The SEER data does not capture specific comorbidities, performance status, or pulmonary function, which could affect the decision to provide adjuvant chemotherapy. The data of chemotherapy regimen could not provide in this study either which might influent the effect of adjuvant chemotherapy. Despite these limitations, our findings contribute to the treatment strategy for these pathological N0 NSCLC patients.

In conclusion, adjuvant chemotherapy had a benefit against pN0 NSCLC when the tumor size was larger than 4 cm. Its effect on tumors between 3 and 4 cm was not remarkable. Poorly-differentiated NSCLC was still a high-risk factor in the pT2N0 stage, and adjuvant chemotherapy provided a benefit in such cases.

## Consent to participate

Informed consent from all participants was waived with the understanding that the released information would be used strictly for research purposes.

## Data Availability

The datasets generated during and/or analyzed during the current study are available from the corresponding author on reasonable request.
